# SARS-CoV-2 XBB.1.5 infects wild-type C57BL/6 mice and induces a protective CD4^+^ T cell response required for viral clearance

**DOI:** 10.3389/fcimb.2025.1621226

**Published:** 2025-08-01

**Authors:** Amany Elsharkawy, Chinonye Dim, Chunyu Ge, Lila D. Patterson, Zainab Nabi, Mukesh Kumar

**Affiliations:** ^1^ Department of Biology, College of Arts and Sciences, Georgia State University, Atlanta, GA, United States; ^2^ Center of Diagnostics and Therapeutics, Georgia State University, Atlanta, GA, United States; ^3^ Vascular Biology Center, Medical College of Georgia at Augusta University, Augusta, GA, United States

**Keywords:** SARS-CoV-2, omicron, XBB.1.5, C57BL/6 mice, CD4^+^ T cell, CD8^+^ T cell

## Abstract

Mouse models are critical for studying SARS-CoV-2 pathogenesis and evaluating therapeutic and preventive strategies. Standard C57BL/6 mice are generally resistant to infection with the ancestral SARS-CoV-2 strain due to inefficient binding of the viral spike protein to the murine angiotensin-converting enzyme 2 (ACE2) receptor. Although human ACE2 transgenic mice can support robust pulmonary infection, these models often develop fatal encephalitis, a pathology not commonly observed in humans. We and others have previously shown that certain SARS-CoV-2 variants can infect wild-type C57BL/6 mice and cause discernible disease. However, the susceptibility of C57BL/6 mice to recently emerged Omicron subvariants, and the role of T cell-mediated immunity in controlling these infections, remain incompletely understood. Herein, we evaluated the susceptibility of wild-type C57BL/6 mice to infection with the SARS-CoV-2 Omicron subvariant XBB.1.5. We assessed viral burden, innate and adaptive immune responses, and virus-induced lung pathology. Our findings demonstrate that XBB.1.5 efficiently replicates in both the upper and lower respiratory tracts of C57BL/6 mice, inducing significant lung inflammation and pathology. Infection elicited a robust pulmonary CD4^+^ and CD8^+^ T cell response. Through antibody-mediated depletion studies, we further show that CD4^+^ T cells are critical for viral clearance, particularly in the nasal airways, as their depletion resulted in persistent viral RNA in the upper respiratory tract. These findings underscore the importance of CD4^+^ T cell responses in controlling XBB.1.5 infection and provide a valuable model for studying variant-specific immune responses and pathogenesis.

## 1 Introduction

The SARS-CoV-2 Omicron variant has emerged as the most heavily mutated variant of concern to date, exhibiting substantial alterations across the spike protein that confer enhanced transmissibility and pronounced immune evasion capabilities (Lyke et al., 2022; Pérez-Then et al., 2022; Shuai et al., 2023). Among the various Omicron sublineages, the XBB subvariant arose as a recombinant of two distinct BA.2 derivative lineages, combining mutations that further enhanced viral fitness (Tamura et al., 2023). The XBB.1.5 subvariant has attracted particular attention due to the acquisition of the F486P substitution in the spike protein, a mutation that improves binding affinity to the angiotensin-converting enzyme 2 (ACE2) receptor while simultaneously enhancing resistance to neutralizing antibodies (Yue et al., 2023). As a result, XBB.1.5 demonstrates superior transmissibility and immune escape compared to earlier XBB and other Omicron sublineages (Wang et al., 2023). In recognition of the significant public health risk posed by XBB.1.5, the U.S. Food and Drug Administration (FDA) authorized the deployment of a monovalent XBB.1.5-targeted mRNA vaccine booster in September 2023, aiming to improve vaccine effectiveness against circulating strains (Patel et al., 2023; Wang et al., 2024).

Mouse models are indispensable tools for studying SARS-CoV-2 pathogenesis and for preclinical evaluation of antiviral therapies and vaccine strategies (Ávila-Nieto et al., 2024; Dong et al., 2022; Stone et al., 2025). The human ACE2 transgenic mouse model (K18-hACE2), which expresses the human ACE2 receptor under the cytokeratin-18 promoter, has been widely used to model SARS-CoV-2 infection. Infection of K18-hACE2 mice with the ancestral B.1 strain and several variants of concern, including Alpha (B.1.1.7), Beta (B.1.351), and Delta (B.1.617.2) results in severe pulmonary disease and widespread viral dissemination to the central nervous system, culminating in fatal encephalitis (Jahantigh et al., 2025; Kumari et al., 2021; Natekar et al., 2022; Oh et al., 2024). In contrast, infection with earlier Omicron variants, such as B.1.1.529, typically leads to attenuated disease in K18-hACE2 mice (Halfmann et al., 2022; Natekar et al., 2022). However, recent data have demonstrated that the XBB.1.5 subvariant replicates to high titers and induces severe lung pathology in K18-hACE2 mice (Elsharkawy et al., 2024), suggesting distinct pathogenic features compared to earlier Omicron sublineages.

Wild-type C57BL/6 mice, on the other hand, are naturally resistant to infection with the ancestral SARS-CoV-2 strain due to inefficient binding between the viral spike protein and the murine ACE2 receptor. Nevertheless, the emergence of variants harboring the N501Y spike mutation, including B.1.1.7, B.1.351, and B.1.617.2 has enabled productive infection in C57BL/6 mice, characterized by high viral loads and elevated inflammatory cytokine and chemokine responses in the lungs (Kar et al., 2024; Pan et al., 2021; Stone et al., 2021). Whether the Omicron XBB.1.5 subvariant can similarly establish efficient infection and induce pathology in wild-type C57BL/6 mice remains unknown. Understanding these variant-specific differences in replication kinetics and tissue tropism is crucial for evaluating the risk posed by emerging variants and for guiding the development of more effective countermeasures.

Despite the clinical relevance of XBB.1.5, detailed studies examining its pathogenicity, tissue tropism, and host immune responses, particularly in widely used mouse models such as wild-type C57BL/6 mice, remain limited. Furthermore, the relative contributions of adaptive immune components, particularly T cell subsets, to the control of XBB.1.5 infection have not been thoroughly elucidated. In the current study, we investigated the susceptibility of wild-type C57BL/6 mice to infection with the SARS-CoV-2 Omicron subvariant XBB.1.5. We quantified viral burden in the upper and lower respiratory tracts using both RT-qPCR and plaque assays. To evaluate virus-induced pathology, we performed histological analysis and assessed immune cell infiltration in the lungs of infected mice. Adaptive immune responses in the lungs and spleen were characterized by flow cytometric analysis following XBB.1.5 infection. Furthermore, to delineate the contribution of T cell subsets to viral control, we employed antibody-mediated depletion of CD4^+^ and CD8^+^ T cells and assessed the impact on viral replication and clearance within the respiratory tract.

## 2 Materials and methods

### 2.1 Cells

Vero E6-TMPRSS2-T2A-ACE2 cells (BEI NR-54970) were cultured in Dulbecco’s modified Eagle medium (DMEM) supplemented with 10% fetal bovine serum (FBS) and 1% penicillin-streptomycin at 37°C (Elsharkawy et al., 2024).

### 2.2 *In vivo* mouse experiments

The SARS-CoV-2 Omicron subvariant hCoV-19/USA/MD-HP40900/2022 (XBB.1.5; NR-59104) was obtained from BEI Resources. Virus stocks were propagated in Vero E6-TMPRSS2-T2A-ACE2 cells as previously described (Elsharkawy et al., 2024). All animal experiments were conducted under biosafety level 3 (ABSL-3) containment at Georgia State University, following protocols approved by the Institutional Animal Care and Use Committee (IACUC Protocol Number: A24003). Wild-type C57BL/6 mice (JAX#0664) were purchased from The Jackson Laboratory. Six-week-old male and female mice were intranasally inoculated under anesthesia with 10^5^ plaque-forming units (PFU) of SARS-CoV-2 XBB.1.5 in phosphate-buffered saline (PBS), or with PBS alone for mock-infected controls. Mice were monitored daily for changes in body weight and clinical signs of disease. At designated time points post-infection, animals were euthanized, and respiratory tissues including nasal turbinates and lungs were collected for downstream virological, immunological, and histopathological analyses (Stone et al., 2025).

### 2.3 Plaque assay

Frozen lung tissues were weighed and homogenized using a Fisherbrand™ Bead Mill 24 Homogenizer (Fisher scientific, catalog# 15-340-163). Virus titers in tissue homogenates were measured by plaque assay using Vero-hACE2-hTRMPSS2 cells as previously described (Rothan et al., 2022).

### 2.4 RNA extraction and quantitative RT-PCR

Nasal turbinates and lung tissues were lysed in RLT buffer. Total RNA was extracted using a Qiagen RNeasy Mini kit (Qiagen, Germantown, MD, USA). Viral RNA levels were measured using SARS-CoV-2 nucleocapsid gene primers (Integrated DNA Technologies, catalog#10006713) and SsoAdvanced universal probe supermix (BIO-RAD, catalog# 1725284). Viral genome copies were calculated using a standard curve and expressed viral genome copies per μg of total RNA (Jahantigh et al., 2025; Oh et al., 2024). Expression of host genes (**Table 1**) were determined using a SsoAdvanced™ Universal SYBR^®^ Green Supermix (BIO-RAD, catalog# 1725271). Fold change for genes in virus-infected samples was calculated against mock-infected samples after normalization to the *Gapdh* gene (Auroni et al., 2023).

**Table 1:** Primer sequences used for RT-qPCR.

Gene (Accession No.)	Forward Primer Sequence (5’->3’)	Reverse Primer Sequence (5’->3’)
*Irf7* (NM_016850.3)	CCCCAGGATCATTTCTGGCA	AGGGTTCCTCGTAAACACGG
*Cxcl10* (NM_021274)	GGTCTGAGTCCTCGCTCAAG	GTCGCACCTCCACATAGCTT
*Ccl2* (NM_011333)	TCACCTGCTGCTACTCATTCACCA	TACAGCTTCTTTGGGACACCTGCT
*Zbp1* (NM_001139519)	GGCAGAAGCTCCTGTTGACT	CTGTCCTCCTTCTTCAGGCG

### 2.5 Immunohistochemistry

Lung tissue sections were stained with hematoxylin and eosin (H&E) for histopathological evaluation (Abcam, catalog# ab245880) (Kumari et al., 2021). For immunofluorescence staining, tissue sections were incubated with DsRNA (J2) mouse antibody (Absolute Antibody, catalog# Ab01299-2.0) overnight at 4°C, followed by Alexa Fluor 594 Goat anti-Mouse IgG (Life Technologies, catalog# A11005) antibodies for 30 min at room temperature. We acquired the images using a Zeiss LSM980 confocal microscope with ×10 (tiles) and ×20 objectives, then analyzed them using the ZEN 3.8 Blue software (Zhang et al., 2023). Lung tissue sections were also stained with CD45-Alexa Fluor^®^ 488 (Cell Signaling Technology, catalog# 59572) and Anti-Actin *α*-Smooth Muscle-Cy3™ antibodies (Sigma, catalog# C6198) overnight at 4°C (Stone et al., 2025). Additionally, lung tissue sections were stained with CD68-Alexa Fluor^®^ 488 (Cell Signaling Technology, catalog# 51644) and Anti-Actin *α*-Smooth Muscle-Cy3™ antibodies (Sigma, catalog# C6198) overnight at 4°C. We mounted the stained sections with ProlongTM Glass Antifade Mountant with NucBlue™ StainDAPI (Thermo Fisher Scientific, catalog# P36981). Images were acquired with the Invitrogen™- EVOS™ M5000 Cell Imaging System.

### 2.6 Flow cytometry analysis

Spleen and lung single-cell suspensions were generated using the gentle MACS tissue dissociator (Miltenyi Biotec, catalog# 130-093-235). We incubated single-cell suspensions with Fc Block antibody (BD Pharmingen) in BD FACS™ Pre-Sort Buffer (BD Biosciences) for 10 min at room temperature. Cell suspensions from infected lung and spleen tissues were incubated with antibodies against the following markers: FITC Rat Anti-Mouse CD45 (BDB553080), APC Rat Anti-Mouse CD3 (BDB565643), PE Rat Anti-Mouse CD4 (BDB553730), PerCP-Cy5.5 Rat Anti-Mouse CD8β (BDB567597), and fixable Viability Stain 575V (BD Biosciences). Flow cytometry data was acquired on a BD LSRFortessa™ Cell Analyzer and was analyzed with the FlowJo software (Roe et al., 2012).

### 2.7 Depletion studies

To deplete CD4^+^ or CD8^+^ T cell populations, mice were administered intraperitoneal injections of monoclonal antibodies (mAbs) targeting either CD4 or CD8β. Specifically, 100 μL of anti-mouse CD4 mAb (clone GK1.5, IgG2b; BioXcell) or anti-mouse CD8β mAb (clone 53–5.8, IgG1; BioXcell) was administered at a dose of 200 μg/mouse. To confirm depletion of targeted cells, splenic cell suspensions were incubated with antibodies against the following markers: FITC Rat Anti-Mouse CD45 (BDB553080), PE-Cy™7 Anti-Mouse CD3 (BDB552774), PE Rat Anti-Mouse CD4 (BDB553730), PerCP-Cy5.5 Rat Anti-Mouse CD8β (BDB567597) following the protocol described above. Treatments were performed on days -3 and -1 prior to XBB.1.5 infection and on days 1, 3, 6, and 12 post-infection. Control animals received 100 μL of PBS via intraperitoneal injection following the same schedule. The CD8β-specific antibody (clone 53–5.8) has been validated to achieve complete depletion of CD8^+^ T cells, while the CD4-specific antibody (clone GK1.5) effectively binds to and depletes CD4^+^ T cells by competitive binding to the CD4 molecule. Successful depletion using these antibodies has been confirmed in multiple prior studies (Ferrari de Andrade et al., 2020; Hollern et al., 2019; Shi et al., 2024).

### 2.8 Statistical analysis

We performed the statistical analyses using the GraphPad Prism software, version 10 and considered results statistically significant at P-values of *p* < 0.05. Statistical tests are indicated in figure legends.

## 3 Results

### 3.1 XBB.1.5 efficiently replicates in the upper and lower respiratory tract of C57BL/6 mice

**Figure 1:**
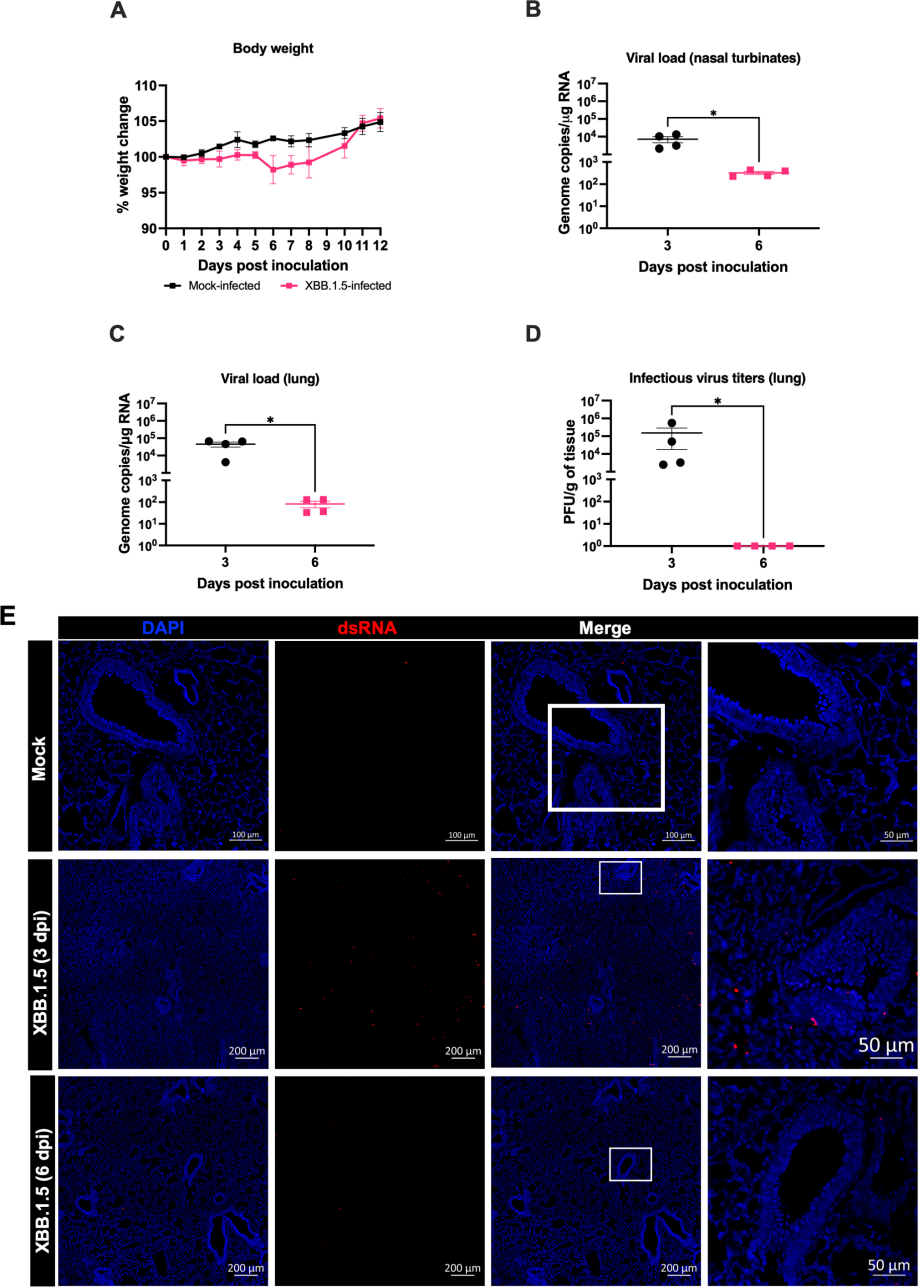
SARS-CoV-2 XBB.1.5 replicates in the upper and lower respiratory tract of wild-type C57BL/6 mice. Six-week-old male and female C57BL/6 mice were intranasally inoculated with 10^5^ PFU of SARS-CoV-2 XBB.1.5 or PBS (mock-infected controls). **(A)** Body weight was monitored daily for up to 12 days post-inoculation (dpi) (n = 5 for XBB.1.5-infected mice; n = 4 for mock-infected mice). Body weights are expressed as the percentage of the initial weight at day 0. Data represent mean ± SEM. **(B, C)** Viral RNA levels in nasal turbinates **(B)** and lungs **(C)** were quantified by RT-qPCR and expressed as genomic copies per microgram of RNA on a logarithmic scale. **(D)** Infectious viral titers in lung tissues were measured by plaque assay and expressed as PFU per gram of tissue. Statistical significance was determined using an unpaired t-test or Mann–Whitney U test as appropriate (*p < 0.05). Each data point represents an individual mouse, with bars indicating mean ± SEM. **(E)** Immunofluorescence staining of lung sections showing double-stranded RNA (dsRNA; red) and nuclei (DAPI; blue) from mock- and XBB.1.5-infected mice. Representative images from each group are shown (n = 4 animals per group).

To assess the susceptibility of C57BL/6 wild-type mice to the SARS-CoV-2 Omicron subvariant XBB.1.5, six-week-old mice were intranasally inoculated with 10^5^ PFU of XBB.1.5. Following infection, mice were monitored daily for clinical signs of disease and body weight changes for up to 12 days post-inoculation (dpi). Throughout the observation period, XBB.1.5-infected mice exhibited no significant weight loss and did not develop severe clinical symptoms (**Figure 1A** **),** suggesting that infection with this variant results in a mild disease phenotype in this mouse model.

In independent experiments, mice were intranasally inoculated with 10^5^ PFU of the XBB.1.5 variant and euthanized at 3 and 6 dpi for analysis. Viral RNA levels were quantified by RT-qPCR. High viral RNA loads were detected in the nasal turbinates at 3 dpi (mean = 7.2 × 10³ copies), followed by a significant reduction at 6 dpi (mean = 3.2 × 10² copies) (**Figure 1B** **).** Similarly, viral RNA levels in the lungs were elevated at 3 dpi (mean = 4.5 × 10^4^ copies) but declined markedly by 6 dpi (mean = 8.2 × 10¹ copies) (**Figure 1C** **).** In addition, plaque assay analysis confirmed the presence of infectious virus in lung homogenates at 3 dpi (mean = 1.5 × 10^5^ PFU). However, no infectious virus was detected in the lungs by 6 dpi (**Figure 1D** **).**

During viral infection, the accumulation of double-stranded RNA (dsRNA) serves as a hallmark of active viral replication (Li et al., 2021). To assess viral replication within lung tissues, we performed immunofluorescence staining to detect dsRNA. Consistent with the viral loads quantified by RT-qPCR and plaque assay, lungs from XBB.1.5-infected mice exhibited a markedly increased dsRNA signal compared to mock-infected controls at 3 dpi that waned by 6 dpi (**Figure 1E** **).** These findings further corroborate active viral replication in the pulmonary tissues of C57BL/6 wild-type mice following XBB.1.5 infection.

### 3.2 Inflammatory response and lung pathology following XBB.1.5 infection

**Figure 2:**
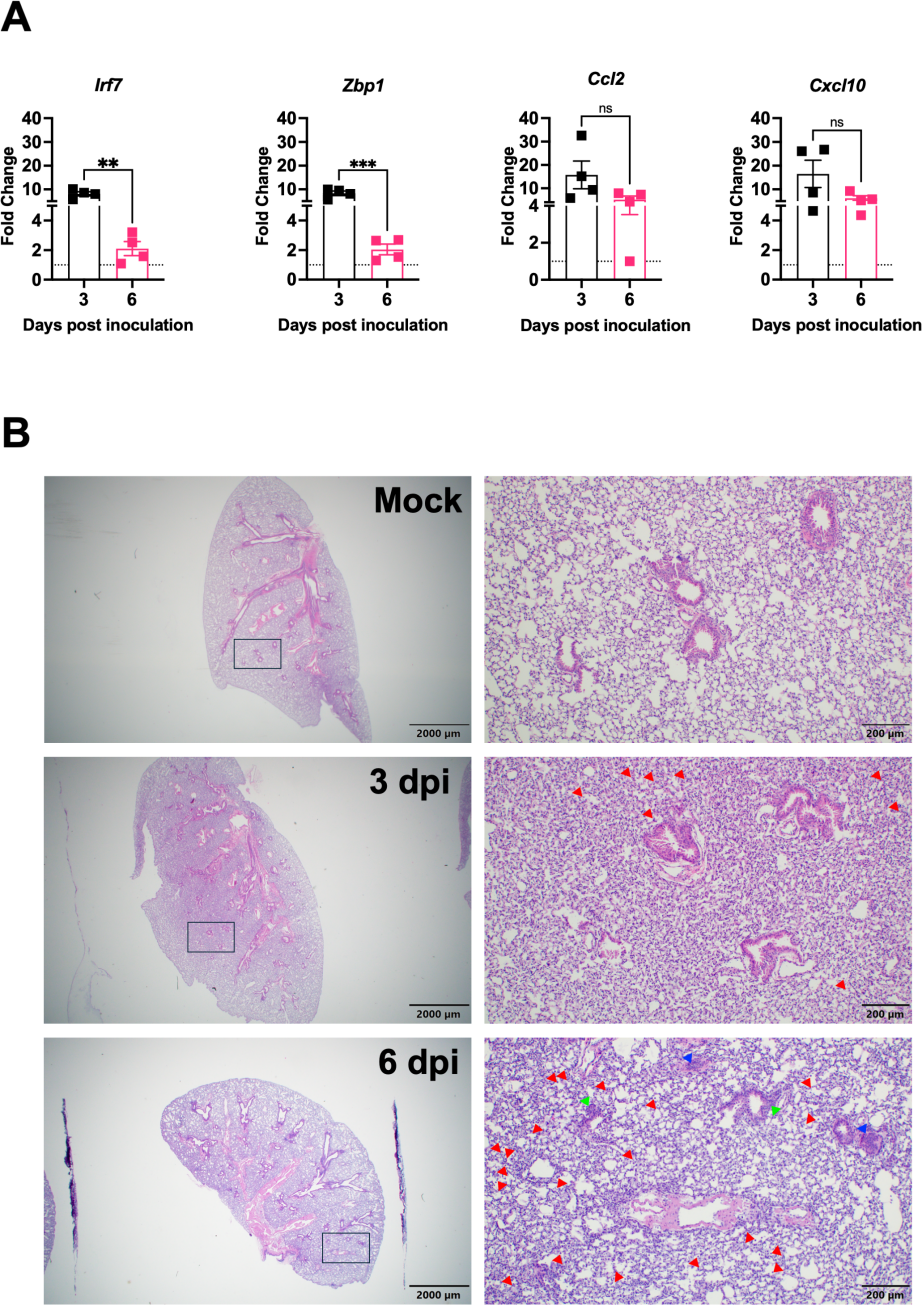
Lung inflammation and pathology following SARS-CoV-2 XBB.1.5 infection in C57BL/6 mice. **(A)** RNA transcript levels of indicated inflammatory and immune-related genes were quantified from lung tissues by RT-qPCR at 3 and 6 dpi (n = 4 animals per group). Data are presented as mean ± SEM. (** p < 0.01; *** p < 0.001; ns represents no significance). **(B)** Representative H&E-stained lung sections collected at 3 and 6 dpi. Images depict low-magnification (scale bar: 2,000 μm) and high-magnification (scale bar: 200 μm) views. Red arrowheads highlight immune cell infiltration into alveolar spaces, green arrowheads indicate bronchial wall thickening, and blue arrowheads denote vascular congestion. One representative image is shown for each group (n = 4 animals per group).

To characterize the host immune response to XBB.1.5 infection, we quantified the expression of key inflammatory genes in lung tissue using RT-qPCR. *Irf7*, a central regulator of the type I interferon response (Qing and Liu, 2023), was upregulated 8.9-fold at 3 dpi and 2.4-fold at 6 dpi compared to mock-infected controls. It was reported that *Zbp1* expression was upregulated in the lungs following infection with major SARS-CoV-2 variants and contributed to lung inflammation and immune cell infiltration (Elsharkawy et al., 2025). Similarly, *Zbp1* showed an 8.3-fold increase at 3 dpi and decreased significantly to 2.0-fold by 6 dpi in XBB.1.5-infected lungs. *Ccl2*, previously reported to be elevated in SARS-CoV-2-infected lungs (Ranjbar et al., 2022), showed a 19-fold increase at 3 dpi and remained elevated (mean = 7.9-fold) at 6 dpi. Expression of *Cxcl10*, a biomarker associated with severe COVID-19 outcomes (Gudowska-Sawczuk and Mroczko, 2022), was also significantly induced, with 20.4-fold and 6.2-fold increases at 3 and 6 dpi, respectively (**Figure 2A** **).**

To assess virus-induced lung pathology following XBB.1.5 infection, histological examination of lung tissues was performed. Hematoxylin and eosin (H&E) staining revealed significant pathological alterations in infected lungs as early as 3 dpi. Compared to mock-infected controls, lungs from infected mice exhibited marked immune cell infiltration into perivascular and alveolar regions, consolidation of alveolar spaces, and evidence of vascular congestion (**Figure 2B**). These histopathological changes are indicative of acute lung injury and reflect robust inflammatory responses induced by XBB.1.5 infection.

### 3.3 Pulmonary immunopathology induced by XBB.1.5 variant in C57BL/6 mice

**Figure 3:**
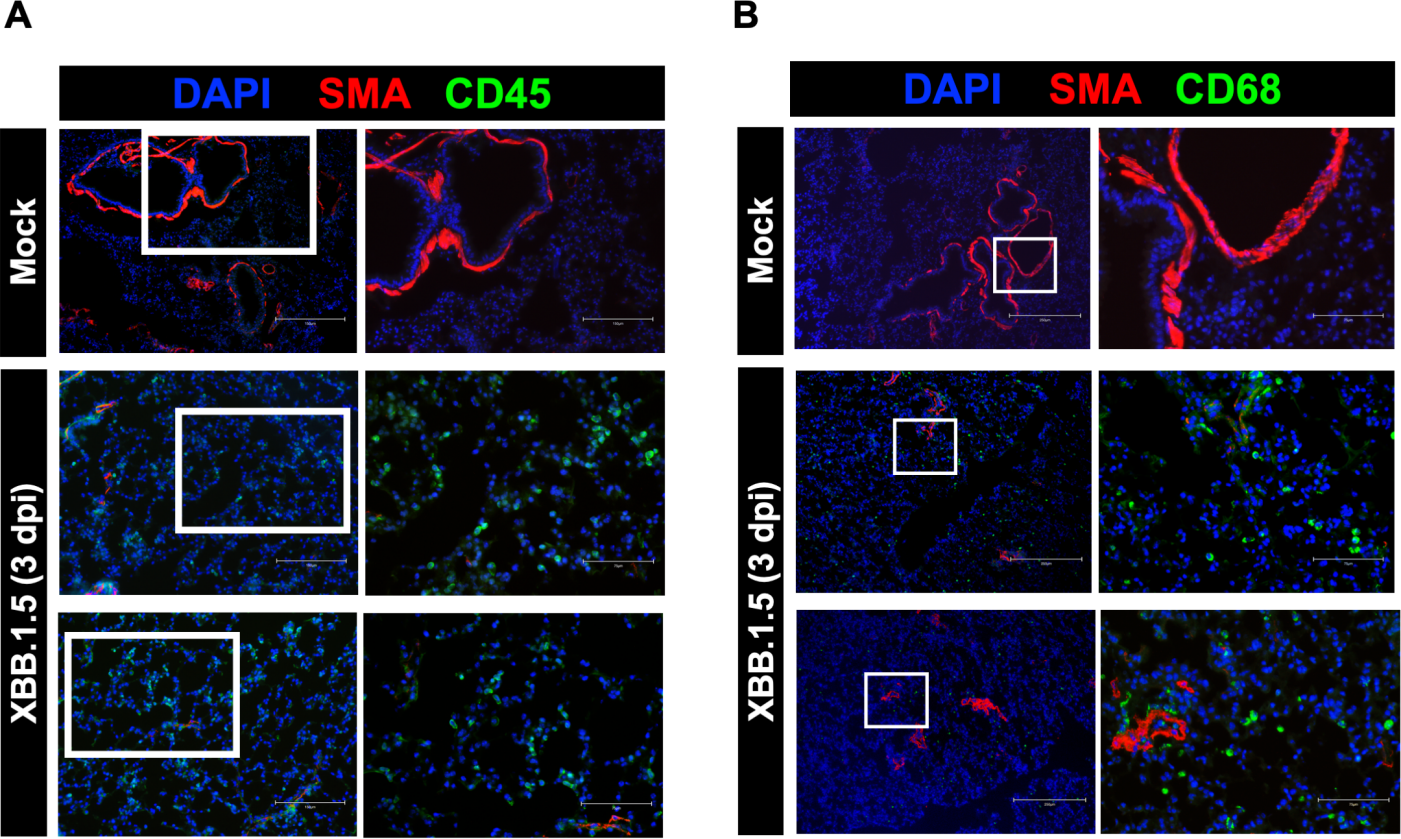
Immunopathology in the lungs of C57BL/6 mice following SARS-CoV-2 XBB.1.5 infection. **(A)** Representative immunofluorescence images of lung sections from mock- and XBB.1.5-infected mice stained with CD45-Alexa Fluor^®^ 488 (green, leukocytes), Anti-Actin α-Smooth Muscle-Cy3™ (red, smooth muscle), and DAPI (blue, nuclei). Scale bars: 150 μm (low magnification) and 75 μm (high magnification). **(B)** Representative immunofluorescence images of lung sections stained with CD68-Alexa Fluor^®^ 488 (green, macrophages), Anti-Actin α-Smooth Muscle-Cy3™ (red, smooth muscle), and DAPI (blue, nuclei). Scale bars: 250 μm (low magnification) and 75 μm (high magnification). n = 4 animals per group.

Previous studies have reported elevated levels of CD45^+^ immune cells in the lungs of COVID-19 patients (Won et al., 2022). To assess immune cell infiltration in response to XBB.1.5 infection, we performed immunofluorescence staining for CD45. Compared to mock-infected controls, XBB.1.5-infected mice exhibited a marked increase in CD45^+^ cells in the lungs at 3 dpi (**Figure 3A**). Similarly, postmortem analyses of COVID-19 patients have revealed increased numbers of CD68^+^ macrophages in the lungs (Barbosa et al., 2022; Cao et al., 2022), and their activation has been implicated in pulmonary tissue damage (Ziablitsev et al., 2023). To investigate macrophage infiltration, we performed immunofluorescence staining for CD68. Our data showed a substantial accumulation of CD68^+^ macrophages in the lungs of XBB.1.5-infected mice compared to controls (**Figure 3B** **).** These cells were primarily located in the alveolar spaces, with some clusters also observed around blood vessels. Collectively, these findings indicate that XBB.1.5 infection induces robust immune cell infiltration in the lungs of C57BL/6 wild-type mice.

### 3.4 XBB.1.5 infection elicits a robust pulmonary T cell response

**Figure 4:**
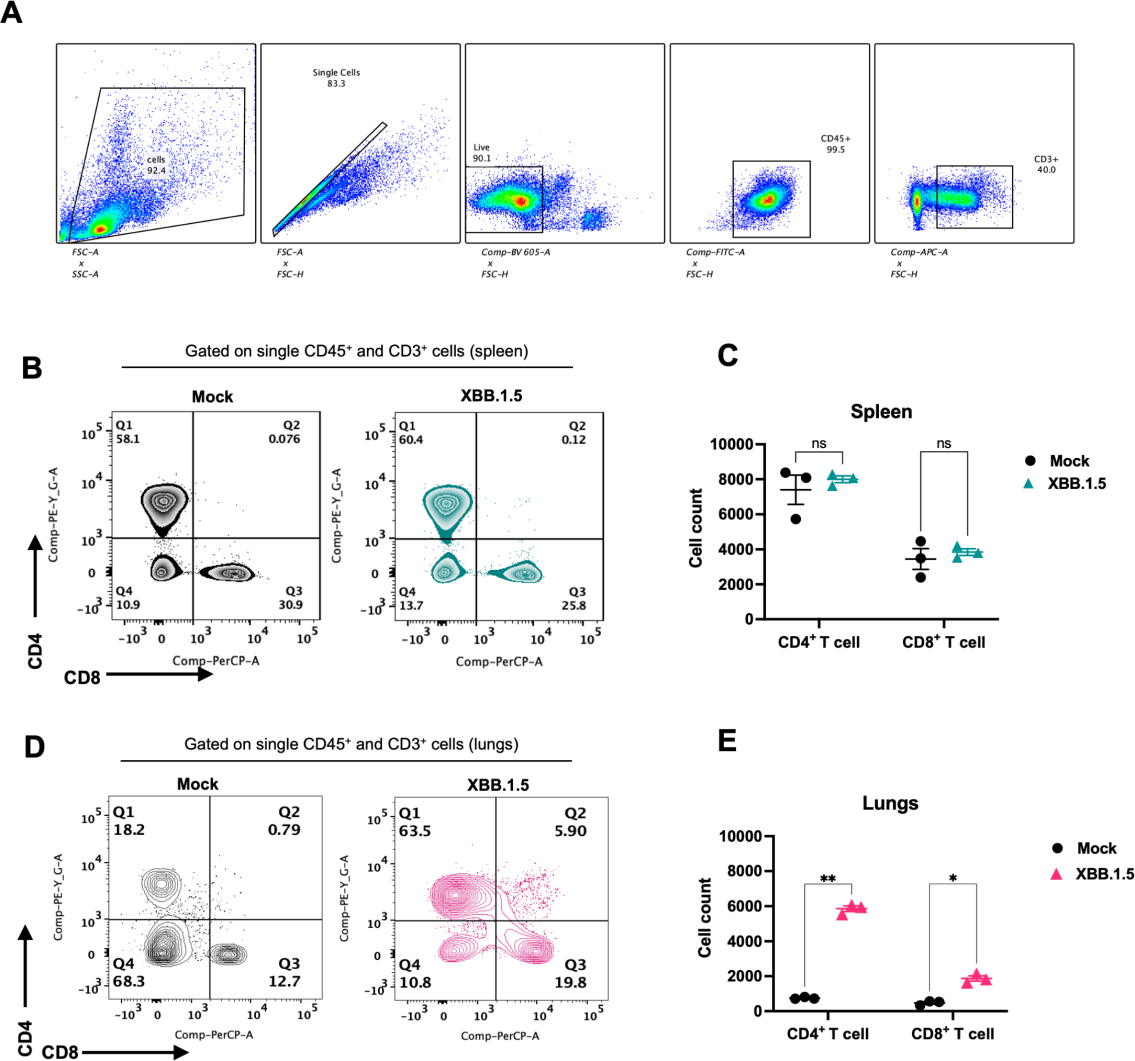
SARS-CoV-2 XBB.1.5 infection induces a robust T cell response in the lungs of C57BL/6 mice. **(A)** Gating strategy for identification of CD4^+^ and CD8^+^ T cells using FlowJo software. **(B)** Representative flow cytometric plots showing expression of CD4 and CD8 visualized by surface staining on CD45^+^ CD3^+^ cells in mock- and XBB.1.5-infected spleens. **(C)** Quantification of CD4^+^ and CD8^+^ T cell numbers in the spleen. **(D)** Representative flow cytometric plots showing surface expression of CD4 and CD8 on CD45^+^CD3^+^ cells in lungs from mock- and XBB.1.5-infected mice. **(E)** Quantification of CD4^+^ and CD8^+^ T cell numbers in the lungs. Each data point represents an individual mouse (n = 3 per group), with bars indicating mean ± SEM. Statistical significance was assessed using two-way ANOVA followed by Šídák’s multiple comparisons test (*p < 0.05; **p < 0.01; ns represents no significance).

Early and robust T cell responses have been associated with effective viral control in mild cases of COVID-19, whereas impaired T cell responses are often observed in severe cases (Le Bert et al., 2020; Tan et al., 2021). To characterize the cellular immune response in XBB.1.5-infected mice, we performed flow cytometric analysis on spleen and lung homogenates at 6 dpi. The gating strategy is presented in **Figure 4A**. No significant differences were detected in the frequencies of splenic CD4^+^ and CD8^+^ T cells between mock- and XBB.1.5-infected mice (**Figures 4B, C**). In contrast, a significant increase in lymphoid cell populations was observed in the lungs of XBB.1.5-infected mice. Specifically, the mean number of CD4^+^ T cells was 5,859 in infected lungs compared to 748 in mock-infected controls, and the mean number of CD8^+^ T cells was 1,874 compared to 474 in mock-infected lungs (**Figures 4D, E**).

### 3.5 Essential role of CD4^+^ T cells in mediating viral clearance from the upper respiratory tract

**Figure 5:**
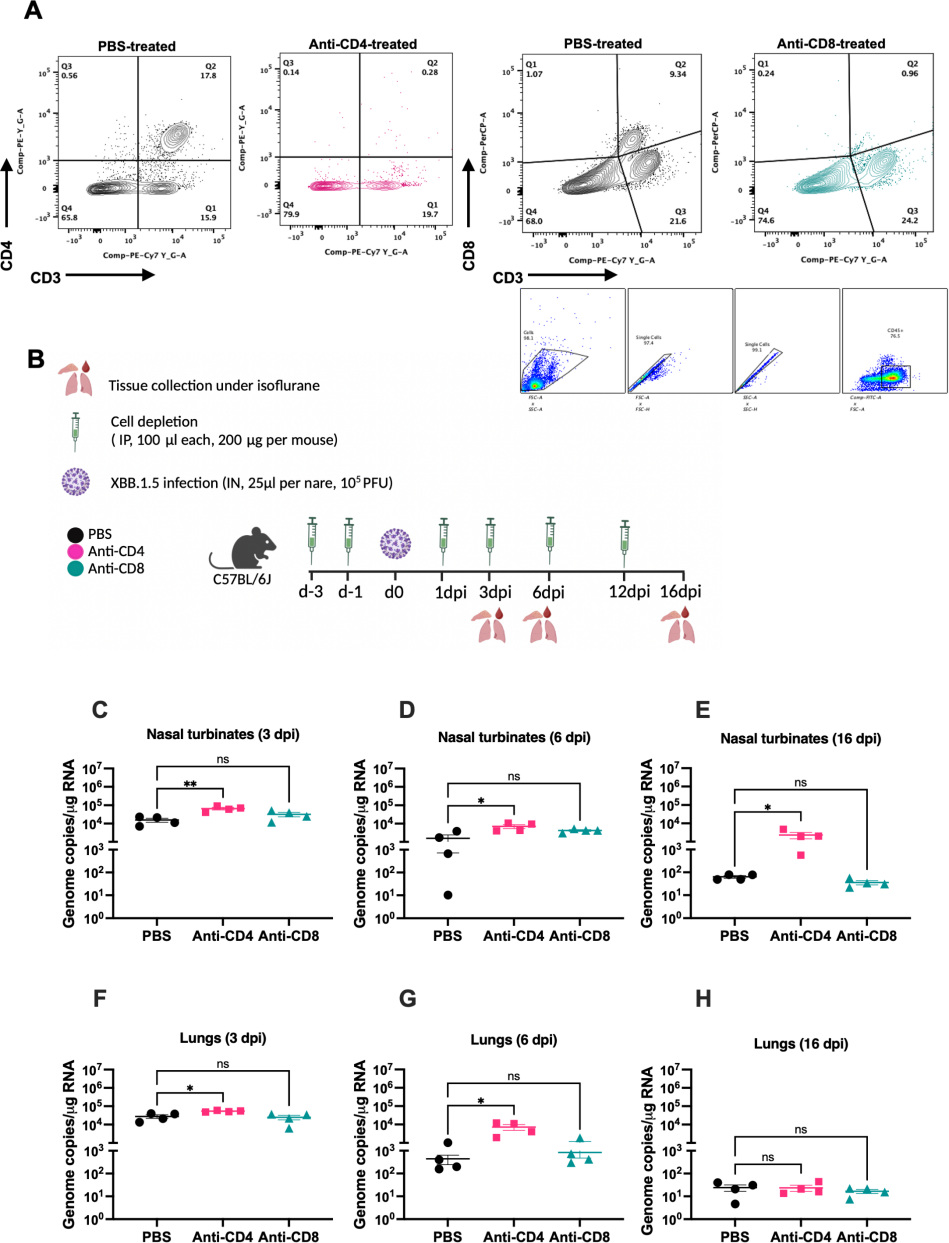
CD4^+^ T cells are required for viral clearance from the upper respiratory tract following SARS-CoV-2 XBB.1.5 infection. **(A)** Gating strategy and FACS plots of CD4^+^ and CD8^+^ T cells in splenic tissues of PBS- and Anti-CD4- or Anti-CD8-treated mice. Representative FACS plots are shown (n=4 per group). **(B)** Schematic overview of the study design. C57BL/6 mice were depleted of either CD4^+^ or CD8^+^ T cells by intraperitoneal administration of 200 μg of anti-mouse CD4 or anti-mouse CD8β monoclonal antibodies at days -3, -1, 1, 3, 6, and 12 relatives to infection. **(C–E)** Viral RNA levels in the nasal turbinates at 3, 6, and 16 dpi quantified by RT-qPCR and expressed as genomic copies per microgram of RNA. **(F–H)** Viral RNA levels in the lungs at 3, 6, and 16 dpi, similarly quantified. Each point represents an individual mouse (n = 4 per group), with data shown as mean ± SEM. Statistical significance was assessed using ordinary one-way ANOVA followed by Dunnett’s multiple comparisons test, comparing depletion groups to the PBS-treated control group (*p < 0.05; **p < 0.01; ns represents no significance).

To assess the role of T cells in viral clearance during SARS-CoV-2 infection, we employed antibody-mediated depletion of CD4^+^ and CD8^+^ T cell populations. To confirm the depletion of targeted cells, spleens were collected a day following PBS, Anti-CD4, and Anti-CD8 intraperitoneal treatment (**Figure 5A**). Flow cytometric analysis confirmed successful depletion of CD4^+^ and CD8^+^ T cells following Anti-CD4 and Anti-CD8 treatment, respectively. XBB.1.5-infected mice were intraperitoneally injected with depleting antibodies (anti-CD4 or anti-CD8; 200 µg/mouse) on days -3 and -1 prior to infection, and subsequently on days 1, 3, 6, and 12 post-infection. As a control, a group of XBB.1.5-infected mice were injected intraperitoneally with 100 μL of PBS following the same schedule (**Figure 5B** **).** Animals were sacrificed at 3-, 6-, and 16 dpi, and nasal turbinates and lung tissues were collected for viral load quantification.

CD4^+^ T cell depletion resulted in a significant increase in viral load in the nasal turbinates at both 3 and 6 dpi compared to PBS-treated controls (**Figures 5C, D**). In contrast, CD8^+^ T cell depletion caused a modest, but not statistically significant, elevation in viral loads at the same time points (**Figures 5C, D**). Importantly, viral RNA persisted in the nasal turbinates of CD4^+^ T cell-depleted mice at 16 dpi (**Figure 5E**), whereas only minimal viral RNA was detectable in CD8^+^ T cell-depleted and PBS-treated mice. In the lungs, CD4^+^ T cell-depleted mice similarly exhibited significantly higher viral loads at 3 and 6 dpi compared to PBS controls (**Figures 5F, G**). CD8^+^ T cell depletion led to a slight, non-significant increase in lung viral loads at 6 dpi (**Figure 5G**). By 16 dpi, viral RNA levels were negligible across all groups in lung tissues (**Figure 5H** **).** These findings indicate that CD4^+^ T cells play a critical role in the clearance of SARS-CoV-2 from the upper respiratory tract.

## 4 Discussion

In this study, we demonstrate that the SARS-CoV-2 Omicron subvariant XBB.1.5 efficiently replicates in both the upper and lower respiratory tracts of C57BL/6 mice, leading to the development of lung inflammation and pathological changes. Furthermore, XBB.1.5 infection elicited a robust adaptive cellular immune response within the lungs. Our findings highlight a critical role for T cell-mediated immunity in controlling viral replication, with CD4^+^ T cells playing a particularly important role in mediating viral clearance in the nasal airways. In contrast, depletion of CD8^+^ T cells had a minimal impact on viral burden and clearance in the respiratory tract, suggesting that CD4^+^ T cells are the primary contributors to antiviral defense in this model. Collectively, these results provide new insights into the pathogenesis and immune control of XBB.1.5 infection in wild-type mice.

Wild-type C57BL/6 mice are generally resistant to infection by the ancestral SARS-CoV-2 strain without viral adaptation. We and others have previously reported that while the original B.1 virus was unable to productively infect C57BL/6 mice, certain variants of concern, such as Alpha (B.1.1.7) and Beta (B.1.351), conferred the capacity for efficient infection and high-titer replication in the murine lungs (Pan et al., 2021; Stone et al., 2021). In the present study, we extend these observations by demonstrating that the Omicron subvariant XBB.1.5 is capable of replicating in both the upper and lower respiratory tracts of wild-type C57BL/6 mice, inducing notable lung inflammation and pathology. Our findings are consistent with recent reports indicating that the XBB.1.5 spike protein exhibits enhanced binding affinity to both human and murine ACE2 receptors (Tachibana et al., 2024; Tamura et al., 2023; Yue et al., 2023). This increased receptor binding likely underpins the observed species tropism expansion, allowing XBB.1.5 to infect murine models without the requirement for additional adaptation. Furthermore, recent studies have shown that select Omicron sublineages, including such as BA.5 and XBB variants replicate more efficiently in hACE2-expressing mice compared to earlier Omicron variants, with enhanced viral titers detected in both the nasal turbinates and lungs (Shuai et al., 2023; Stewart et al., 2023). In line with these variant-specific trends, we recently demonstrated that XBB.1.5 infection in hACE2-transgenic mice results in severe pulmonary pathology, systemic viral dissemination, and substantial mortality (Elsharkawy et al., 2024). Together, these findings suggest that the XBB.1.5 variant possesses distinct biological properties that facilitate broader host range infection and heightened pathogenicity compared to earlier Omicron strains.

Multiple lines of evidence support the critical role of T cell-mediated immunity in modulating disease severity in COVID-19 patients (Guo et al., 2022; Tan et al., 2021). Clinical studies have shown that a reduction in peripheral T cell counts correlates with increased disease severity in symptomatic individuals (Rydyznski Moderbacher et al., 2020). Consistent with these findings, we previously reported that hACE2-expressing mice infected with the XBB.1.5 variant exhibited impaired T cell responses in the lungs, which was associated with severe disease manifestations (Elsharkawy et al., 2024). In the current study, we observed a significant increase in CD4^+^ and CD8^+^ T cell populations in the lungs of C57BL/6 mice following XBB.1.5 infection, indicative of a robust adaptive immune response. Importantly, depletion of CD4^+^ T cells resulted in markedly elevated viral loads in both the lungs and nasal turbinates, underscoring the essential role of CD4^+^ T cells in controlling viral replication. Notably, persistent viral RNA was detected in the nasal turbinates of CD4^+^ T cell-depleted mice, suggesting that CD4^+^ T cells are particularly critical for clearance of virus from the upper respiratory tract. These findings agree with previous observations by Kar et al., who reported prolonged viral replication in the upper respiratory tract of C57BL/6 mice infected with the Beta (B.1.351) variant following T cell depletion (Kar et al., 2024).

Collectively, our findings demonstrate that C57BL/6 mice are susceptible to infection with the SARS-CoV-2 Omicron subvariant XBB.1.5. Infected mice exhibited mild clinical disease, rapid viral clearance, and a robust pulmonary T cell response. Our results further indicate that CD4^+^ T cells, but not CD8^+^ T cells, are essential for controlling viral replication in both the upper and lower respiratory tracts. Moreover, CD4^+^ T cells were specifically required for efficient viral clearance from the nasal airways, underscoring their critical role in mediating antiviral immunity during XBB.1.5 infection.

## Data Availability

The original contributions presented in the study are included in the article/supplementary material. Further inquiries can be directed to the corresponding author.
